# Associations between pre-pandemic authoritative parenting, pandemic stressors, and children’s depression and anxiety at the initial stage of the COVID-19 pandemic

**DOI:** 10.1038/s41598-023-42268-x

**Published:** 2023-09-20

**Authors:** Karina G. Heaton, Nicolas L. Camacho, Michael S. Gaffrey

**Affiliations:** 1 Graduate School of Applied and Professional Psychology, Rutgers University, Piscataway, NJ USA; 2 Department of Psychology and Neuroscience, Duke University, Durham, NC USA; 3 Children’s Wisconsin, Milwaukee, WI USA; 4 Division of Pediatric Psychology and Developmental Medicine, Department of Pediatrics, Medical College of Wisconsin, Milwaukee, WI USA

**Keywords:** Psychology, Human behaviour, Risk factors, Public health, Anxiety, Depression

## Abstract

Large-scale changes due to the Novel Coronavirus (COVID-19) pandemic negatively affected children’s mental health. Prior research suggests that children’s mental health problems during the pandemic may have been concurrently attenuated by an authoritative parenting style and exacerbated by family stress. However, there is a gap in the literature investigating these mechanisms and whether pre-pandemic authoritative parenting had a lasting positive influence on children’s mental health while they were exposed to pandemic-related family stressors. The current study begins to fill this gap by investigating these unique relationships in a sample of 106 4–8 year old children (51% female). Before the pandemic, caregivers completed questionnaires on their parenting style and their children’s depression and anxiety symptoms. Shortly after the onset of COVID-19’s stay-at-home mandate, parents answered questionnaires about their children’s depression and anxiety symptoms and pandemic-related family stressors. Child depression and anxiety symptom severity increased. Higher levels of pandemic-related family stress were associated with increases only in child anxiety scores. Further, greater endorsement of a pre-pandemic authoritative parenting style was associated with smaller changes only in child depression scores. Study findings elucidate unique and complex associations between young children’s anxiety and depression symptoms severity and pre-pandemic parenting and pandemic-related family stressors.

## Introduction

The Novel Coronavirus (COVID-19) pandemic led to many societal changes and has been conceptualized by researchers as a “collective trauma” because of the hundreds of thousands of COVID-19 deaths in the United States, millions of people left unemployed, exacerbation of racial and monetary inequities, significant societal events like stay-at-home orders and school closures, and ambiguity about when it would end, all having a vast psychological impact on mental health^1–4^. Emerging research has indicated that COVID-19 had particularly pronounced effects on youth. Multiple studies have reported depression and anxiety disorder prevalence increases and symptom elevations in school-age children and adolescents during the first year of the pandemic^1,5–10^. Interestingly, previous research suggests that children’s emotional responses to a collective trauma might vary due to individual factors, including the parenting style of their caregivers and the amount of trauma-related stress experienced by their family^11–13^. However, few studies to date have directly addressed this question in young children, leaving a critical knowledge gap in our understanding of COVID-19’s impact on early childhood mental health and the factors influencing it.

As noted above, the potential impact of a collective trauma on child wellbeing is the result of a multifactorial process. A helpful framework for defining and studying the interactions between factors within this process is Bronfenbrenner’s Ecological Systems Theory (BEST)^14–16^, which suggests that an individual’s reaction to a given situation or event is the outcome of many interacting factors (e.g., social policy, caregiving relationships, etc.) both at the time of the event and preceding it^14,15^. Using BEST as a guide, available data suggest that understanding the interplay between different components in the child's microsystem (i.e., factors directly acting on the child) within the broader context of the child’s exosystem (i.e., factors indirectly influencing what experiences are available for a child) and chronosystem (i.e. when events occur)^14–16^ is critical for understanding the effects of COVID-19 on children’s mental health. Perhaps most importantly, BEST also suggests that understanding factors preceding the onset of a stressful life event can provide novel insight into potential actions capable of preventing negative outcomes during similar, future events^14–16^. Thus, given that pandemics similar to COVID-19 have a significant likelihood of occurring in the near future^17^, identifying factors affecting child mental health that preceded and/or occurred during the early stages of the pandemic is of critical public health importance.

One factor in a child’s microsystem that can impact their mental health is parenting style. Parenting style is characterized as parenting behaviors that influence the way parents interact with their child^18,19^. Following Baumrind’s typology^18,19^, authoritative parenting is emotionally supportive, sensitive to a child’s needs, provides rational reasoning behind rules when directing the child’s behavior, sets high standards, gives appropriate autonomy to the child, and facilitates clear, bidirectional communication^20–22^. Since authoritative parenting style provides an atmosphere with these qualities and is a strong predictor of young children’s healthy adjustment and psychosocial competence^23^, experiencing a predominantly authoritative parenting style as a child has been suggested to be a protective factor against the development of internalizing symptoms, such as depression and anxiety, and maladjustment at different stages of socioemotional development and even among the elderly^24–29^. In contrast to the benefits of authoritative parenting, authoritarian parenting provides little nurturance and responsiveness to a child’s needs and restricts the child’s autonomy by instilling high obedience^20,22,23^, and permissive parenting has a lack of demands that do not provide a sufficient environment to guide the child through self-regulation^20^. Because of these qualities, authoritarian and permissive parenting are both largely associated with children’s mental health problems and maladjustment^18,19,22,25,26^.

Despite strong evidence that authoritative parenting style is a protective factor for child mental health problems^24–29^, very few studies have investigated authoritative parenting style’s impact on children’s mental health in the context of collective traumas and/or other large-scale societal stressors. Abu Baker et al.^11^ found that authoritative parenting style was associated with less child mental health problems during the stressful context of traumatic political violence. In addition to Abu Baker et al.^11^, to our knowledge only two studies have investigated the concurrent associations between children’s mental health and parenting style within the context of the COVID-19 pandemic^30,31^. One study conducted in Indonesia found that paternal authoritative parenting style during the pandemic was concurrently associated with lower internalizing symptoms in children aged 3 to 12 years old^30^. Relatedly, a study conducted a few months after China mandated citizens to stay at home found that authoritarian parenting at that time mediated the negative relationship between socioeconomic status and preschoolers’ anxiety^31^. Importantly, these early findings indicate that authoritative parenting may act to attenuate the negative effects of COVID-19 on young child mental health^30^. However, given that parenting measures were collected following the onset of COVID-19 in these studies^30,31^, they are not able to inform whether parenting style measured prior to the pandemic is *predictive of changes* in child mental health following the onset of COVID-19. As a result, the potential of promoting an authoritative parenting style as a public health pandemic-related preparedness step remains unclear. A recent study reporting that pre-pandemic factors (i.e., living with parents and social support) played an important role in attenuating the effects of COVID-19 on the experience of depression and anxiety related symptoms in adolescents further underscores the importance of data informing this question^32^.

In light of prior research^1,6,7,33^, the impact of COVID-19 on child mental health likely also varied as a function of familial stress and disruptions in day-to-day family routines^8,34–38^ at the onset of the pandemic. More specifically, large disruptions in family routines and resultant household chaos due to public health measures in response to COVID-19 have been found to be associated with parental stress and emotional and behavioral difficulties in children^39–41^. School closures in particular, which occurred early in the pandemic, have been noted as having far-reaching, negative effects on caregiver levels of pandemic associated distress^42^ and child loneliness and mental health^1,6,7,10,33,43^. As a result, considering this prior research and BEST, centering investigations both in terms of specific COVID-19 related event (e.g., school closures) and period of reaction following the event (e.g., 3-months) would greatly benefit understanding the influence of external (i.e., exosystem) and time-sensitive (i.e., chronosystem) factors on the association(s) between family-related stressors and young child mental health during the pandemic. Nevertheless, little published data has taken this approach in young children, leaving questions about the association between acute COVID-19 related family stress and child emotion unanswered.

### Present study

The current study capitalized on an ongoing investigation of early childhood mental health started prior to the onset of the COVID-19 pandemic to investigate the effects of pre-pandemic authoritative parenting style and family related stress on young child mental health following the acute and significantly stressful event of the pandemic experienced by all families participating in the study. We hypothesized that (1) child depression and anxiety symptom levels would increase following the onset of the COVID-19 pandemic, (2) increased child anxiety and depression symptom levels would be associated with elevations in pandemic-related family stress, and (3) increased caregiver endorsement of authoritative parenting style prior to pandemic onset would be predictive of smaller changes in child depression and anxiety symptom level scores.

## Methods

### Participants

A total of *N* = 323 children and their parents were recruited from the greater Durham, North Carolina, United States area. Families were recruited through a subject recruitment database maintained by Duke University’s Department of Psychology and Neuroscience and local community events (e.g., children’s museums, farmers market). Parents completed a phone screener between 2018 and 2020. Parents were required to be a biological parent and primary caregiver and must have lived with the child for at least the last 6 months. Exclusion criteria on the phone screener included IQ < 70, developmental delays, premature birth (< 35 weeks gestation), neurological condition (e.g., epilepsy), substance exposure in utero, and psychiatric medication use. In addition, parents were asked about the presence of depressive symptoms in their children using the Preschool Feelings Checklist^44^. To ensure the representation of a wide range of depressive symptoms, children with moderate levels of depressive symptomatology (2 items endorsed) were ineligible to participate.

Seventy-four children were ineligible for this study. Of 249 eligible children, the parents of 17 declined to participate, 47 did not complete the baseline visit, and 14 canceled their baseline visit due to the start of the COVID-19 pandemic. A total of *n* = 171 children completed an initial in-person lab assessment (baseline visit) between November 2018 and March 2020. Parents were contacted two months after North Carolina issued a stay-at-home order due to COVID-19 (March 27th, 2020) and were asked to fill out additional questionnaires. The parents of 127 children replied to the online surveys, but only the parents of 113 children provided data beyond signing the consent form. Participants with complete datasets at the subscale level made up the main sample of this study (*N* = 106). These data were collected from May to July 2020. Table 1 provides descriptive statistics for these participants (*M*_age_ at baseline = 5.99 years, *SD* = 0.93, range: 4.50—8.13 years; *M*_age_ at follow-up = 6.87 years, *SD* = 0.97, range: 5.13–9.01 years). Parents signed a written consent form and children provided verbal assent. Families were compensated with gift cards and toy prizes. The Institutional Review Board at Duke University approved this study. All research was conducted in accordance with the Declaration of Helsinki.

**Table 1:** Demographic characteristics of participants.

Child characteristics	*n*	%		
Sex assigned at birth				
Female	54	51		
Male	52	49		
Race				
Caucasian	77	72.6		
Black	8	7.6		
Asian	3	2.9		
American Indian/Alaska Native	1	0.9		
NHPI	0	0		
Mixed				
Caucasian and Black	5	4.7		
Caucasian and Asian	4	3.8		
Caucasian and NHPI	1	0.9		
Unspecified	7	6.6		
Ethnicity				
Hispanic/Latinx	11	10		
Not Hispanic/Latinx	95	90		
Sibling pairs	13	12.3		

Parent characteristic	Baseline		COVID follow-up	
	*n*	%	*n*	%
Reporters				
Mother	92	87	94	88.7
Father	14	13	9	8.5
Unspecified	–	–	3	2.8

*N* = 106. Demographic characteristics of the children and parents who participated in this study are shown here.

*NHPI* Native Hawaiian/Pacific Islander.

### Procedures

During the baseline visit (T1), parents completed questionnaires about their and their child’s mental and physical health, parent’s parenting styles, their child’s life experiences, and family demographics. Children also completed behavioral and neuroimaging tasks not reported here. The state of North Carolina issued a COVID-19 stay-at-home mandate that resulted in the discontinuation of in-person schooling on March 27th, 2020. Following this order, parents were recontacted and asked to complete a set of online questionnaires about their children’s emotions, mental health symptoms, and family COVID-19 stressors during the first two months following the stay-at-home order (T2). This two-month period was chosen because it was thought to capture a period of acute adjustment and stress in response to COVID-19-related changes in daily life. The average time between the baseline visit and COVID follow-up was 10.62 months (*SD* = 4.49, Range = 2.53–18.37). The ages of the children and their participation timepoints are depicted in Supplementary Figure S1.

### Measures

A summary of the means, standard deviations, and ranges of all the measures used in this study can be found in Table 2. Internal consistency (Cronbach’s α) is noted below. For participants with at least 75% complete item-level data on each rating scale (excluding the Income-to-Needs Ratio), mean scores were calculated using respective item ratings. Additional details of the measures in the study can be found in the Supplementary Materials.

**Table 2:** Means, standard deviations, ranges, and correlation matrix of primary variables of interest.

Variable	*M*	*SD*	Range	1	2	3	4	5	6	7	8	9	10
1. Child Age T1 (years)	5.99	0.93	4.50 – 8.13										
2. Child Age T2 (years)	6.87	0.97	5.13 – 9.01	.92**									
3. ItN T1	3.05	1.13	0.49 – 5.91	.02	.07								
4. LEC T1	0.79	0.71	0 – 3.00	.11	.07	.01							
5. PAS T1	0.43	0.28	0 – 1.39	−.01	−.01	.07	.16						
6. PAS T2	0.64	0.40	0 – 2.00	.14	.11	.03	.10	.50**					
7. PFC-S T1	0.51	0.32	0 – 1.65	−.06	−.07	.16	.15	.47**	.32**				
8. PFC-S T2	0.66	0.40	0 – 1.74	.04	.03	.17	.10	.40**	.58**	.62**			
9. EPII T2	1.24	0.51	0.30 – 2.58	−.05	−.08	−.04	.23*	.03	.39**	.24*	.42**		
10. ATV T1	3.08	0.36	2.19 – 3.93	−.06	−.09	−.19	.01	−.17	−.19	−.21*	−.36**	−.05	
11. P-PANAS NA T1	0.78	0.54	0 – 2.60	.01	−.02	−.02	.11	.16	.16	.21*	.20*	.27**	−.02

*N* = 106. Means, standard deviations, ranges, and Pearson’s correlations among variables of main interest are shown here. T1 = baseline, T2 = COVID Follow-Up. ItN = Income-to-Need ratio, LEC = Life Events Checklist, EPII = Epidemic-Pandemic Impacts Inventory, PAS = Preschool Anxiety Scale, PFC-S = Preschool Feelings Checklist-Scale, ATV = Parenting Practices Questionnaire, Authoritative Style. P—PANAS NA = Positive and Negative Affect Schedule, Negative Affect (Parent).

*Indicates *p* < .05

**Indicates *p* < .01.

#### Income-to-Needs Ratio (ItN)

The ItN^45^ was used as a measure of a family’s socioeconomic status. The income-to-needs ratio was calculated for each family by dividing the family’s income by the poverty level of that year designated to the family’s size. A larger ratio indicates higher socioeconomic status.

#### Life Events Checklist (LEC)

The LEC^46^ was used to measure possible negative life stressors experienced by children before the pandemic (e.g., “Major personal illness or injury.”, “Separation from spouse or partner due to conflict.”, “Death of family member of close friend.”). The LEC has shown acceptable validity and test–retest reliability^46^ as well as utility as a measure of life stress predictive of internalizing symptoms^47^ and peer rejection in youth^48^. Mean effect scores were calculated for each child for events defined as bad that occurred in the past 6 months or earlier. A higher mean score on this scale indicates higher impact experienced by children by a set of early life stressors (α = 0.71).

#### Epidemic Pandemic Impact Inventory (EPII)

The EPII^49^ was used to assess the impact of COVID-19 family stressors during the first two months of the pandemic. While the psychometric properties of the EPII have not been thoroughly investigated due its urgent creation at the start of the COVID-19 pandemic, its promise and utility are underscored by its maintenance in the National Institute of Health Disaster Research Response Repository of COVID-19 Research Tools^50^ and its utility in measuring a wide range of relevant experiences during the pandemic^51^. Caregivers were asked to indicate whether a challenge was experienced. Subsequently, for items endorsed, caregivers reported whether the item had a ‘positive’ or ‘negative’ effect on them (or their family) and to rate how much the experience impacted them (or their family) on a 7-point Likert scale, with higher scores indicating greater impact. Subscales summarized changes due to the pandemic to the individual or family’s Work Employment (e.g., “Laid off from job or had to close own business.”), Education and Training (e.g., “Had a child in home who could not go to school.”), Home Life (e.g., “Difficulty taking care of children in the home.”), Economics (e.g., “Unable to pay important bills like rent or utilities.”), Social Activities (e.g., “Separated from family or close friends.”), Emotional Health and Wellbeing (e.g., "Increase in child's sleep difficulties or nightmares."), Quarantine (e.g., “Isolated or quarantined due to possible exposure to this disease.”), Infection (e.g., “Someone died of this disease while in our home.”), Physical Health (e.g., “Increase in health problems not related to this disease.”), and Positive Changes (e.g., “More quality time with children.”). A single mean score across all subscales summarizing negative effects on the individual or family (i.e., excluding Positive Changes) were calculated. A higher mean score indicates greater family experienced negative impact due to COVID-19 stressors (α = 0.92). Recent work implementing the EPII during the COVID-19 pandemic shows that higher family EPII scores were concurrently associated with worse cognitive and socioemotional well-being^52^ and greater internalizing and externalizing problems in youth^34^.

#### Preschool Anxiety Scale (PAS)

The PAS^53^ was used to measure children’s anxiety symptoms severity before and during the first two months of the COVID-19 pandemic. The factor structure of the PAS aligns with contemporaneous characterizations of anxiety disorders^54^ and indicates that a single score can be calculated to represent overall anxiety symptom severity in young male and female children with acceptable construct validity^53^. Examples of the questions include, “Is afraid of crowded or closed-in places” and “Asks for reassurance when it doesn’t seem necessary.” Mean scores for anxiety were calculated for each child. A higher mean score on the scale indicates greater severity of anxiety symptoms in the child (T1 α = 0.79; T2 α = 0.85).

#### Preschool Feelings Checklist Scale (PFC-S)

The PFC-S^44^ was used to measure children’s depression symptom severity within the past week, before and during the first two months of the COVID-19 pandemic. The PFC-S has shown strong internal consistency in recent work^55^ and is a longer dimensional version of the checklist version with strong psychometric properties^56^. Examples of the questions include, “Appears sad or says s/he feels sad” and “Seems to feel overly guilty.” Mean scores for depression were calculated for each child. A higher mean score on the scale indicates greater severity of depression symptoms in the child (T1 α = 0.83; T2 α = 0.88).

#### Parenting Practices Questionnaire (PPQ)

The PPQ^19^ was used to measure caregivers’ parenting styles (authoritarian, authoritative, permissive) during the baseline visit only. For the purposes of this study, only the authoritative style subscale was used (ATV) because of its role in promoting children’s mental health^24^. The ATV subscale has shown excellent internal consistency and factorial structure in previous work^19^. Examples of the questions include, “Encourages our child to talk about the child's troubles” and “Shows sympathy when our child is frustrated or hurt.” A higher mean score on the scale indicates greater endorsement of an authoritative parenting style (α = 0.86).

#### Positive and Negative Affect Schedule-Parent (P-PANAS)

The P-PANAS^57^ was used to measure parents’ negative affect (NA). In previous work, the P-PANAS evidenced a two-factor structure spanning separate Positive and Negative Affect scales with strong internal consistency, test–retest reliability, and discriminant and convergent validity^57^. This measure was administered to parents during their baseline visit and during the first two months of the COVID-19 pandemic. A higher mean score on the scale indicates a higher presence of parental negative affect on average (α = 0.85). Items asked parents how they have felt over the past week and included words such as “Guilty”, “Scared”, “Hostile”, “Irritable”, and “Ashamed.” Only the scores from the baseline visit are included in analyses to control for pre-pandemic parental negative affect and more clearly interpret the effect of pre-pandemic parenting style on their children’s internalizing symptoms.

### Analytic plan

The final dataset and the scripts that were written to analyze the data are openly available via https://github.com/nicocamacho94/covid_parentingStyle_dep_anx. All analyses were conducted in R version 4.1.2^58^. Details regarding the R packages used here can be found in the Supplementary Materials.

Pearson’s correlations were calculated for all variables of interest. To assess whether there were increases in children’s depression and anxiety severity ratings between the baseline and COVID follow-up timepoints, paired sample t-tests were respectively conducted on the PFC-S and PAS mean scores of the sample with complete datasets (hypothesis 1). Effect size estimates were calculated using Cohen’s *d* and interpretations followed Cohen’s (1988) guidelines of small (0.2 ≤ *d* ≤ 0.5), medium (0.5 ≤ *d* ≤ 0.8), and large (d ≥ 0.8) effect sizes^59^.

Linear regression model assumptions (i.e., residual homoscedasticity and normality) were tested and supported the use of ordinary least squares regression (see Supplementary Materials). Two-tailed hierarchical multiple linear regression analyses were conducted to test the main hypotheses of this study (⍺ = 0.05). To detect multivariate outliers, we performed tests of the Minimum Covariance Determinant (MCD) for each of the final regression models that included the final specific set of included variables. The MCD method estimates both location and scatter and is robust to non-normal variable distributions^60^. Participants outside of the 75th percentile of the MCD distance were considered multivariate outliers for each model^61^ and were excluded from the main regression analyses. For increased specificity, the model predicting depression severity during the pandemic included depression severity before the pandemic and anxiety severity during the pandemic as covariates in step one. Similarly, the model predicting anxiety severity during the pandemic included anxiety severity before the pandemic and depression severity during the pandemic as covariates in step one. Parents' pre-pandemic negative affect was included as a covariate in step one in both models to provide greater specificity to effects between child depression and anxiety symptoms and parenting style. Additional covariates (e.g., age, sex, and prior negative life stressors) were added if they significantly (*p* < 0.05) correlated with symptom severity variables collected during the pandemic.

After controlling for previous and related internalizing symptomatology in both models, the COVID-19 stressors impact variable was entered into the model (hypothesis 2), before the entry of authoritative style in the final step (hypothesis 3). Doing so allowed for the analysis of the individual contributions of each of these variables to the prediction of child anxiety and depression symptom severity and the incremental contribution of pre-pandemic authoritative parenting style to these predictions, independently from the influence of early COVID-19 stressors. At each step, the significance of the inclusion of each variable into the model was assessed using a change in R^2^. Both unstandardized (*b*) and standardized (b*) regression coefficients and adjusted (Adj.) and unadjusted R^2^ are presented. We conducted separate sensitivity analyses to assess the robustness of our results to the inclusion of multivariate outliers, the ItN variable, the LEC variable, and a variable representing the length of time between the baseline and follow-up timepoints. We also estimated cluster-robust standard errors based on family membership to account for any effects of sibling relations in our sample. Details regarding the sensitivity analyses can be found in the Supplementary Tables S1-S8.

## Results

### Bivariate correlations

Table 2 presents the correlations between all variables of interest in this study. Importantly, anxiety and depression symptom severity scores during the pandemic were significantly correlated with each other, and their own and each other’s pre-pandemic scores. They were also significantly correlated with COVID-19 stressors. Depression but not anxiety symptom severity scores during the pandemic were correlated with pre-pandemic authoritative style. Child anxiety and depression symptom severity variables during the pandemic did not significantly differ by biological sex and were not significantly correlated with age, baseline income-to-needs, pre-pandemic negative life events, or time between timepoints. These demographic variables were not included in the primary hierarchical linear regression analyses.

### Paired samples *t*-tests

**Figure 1:**
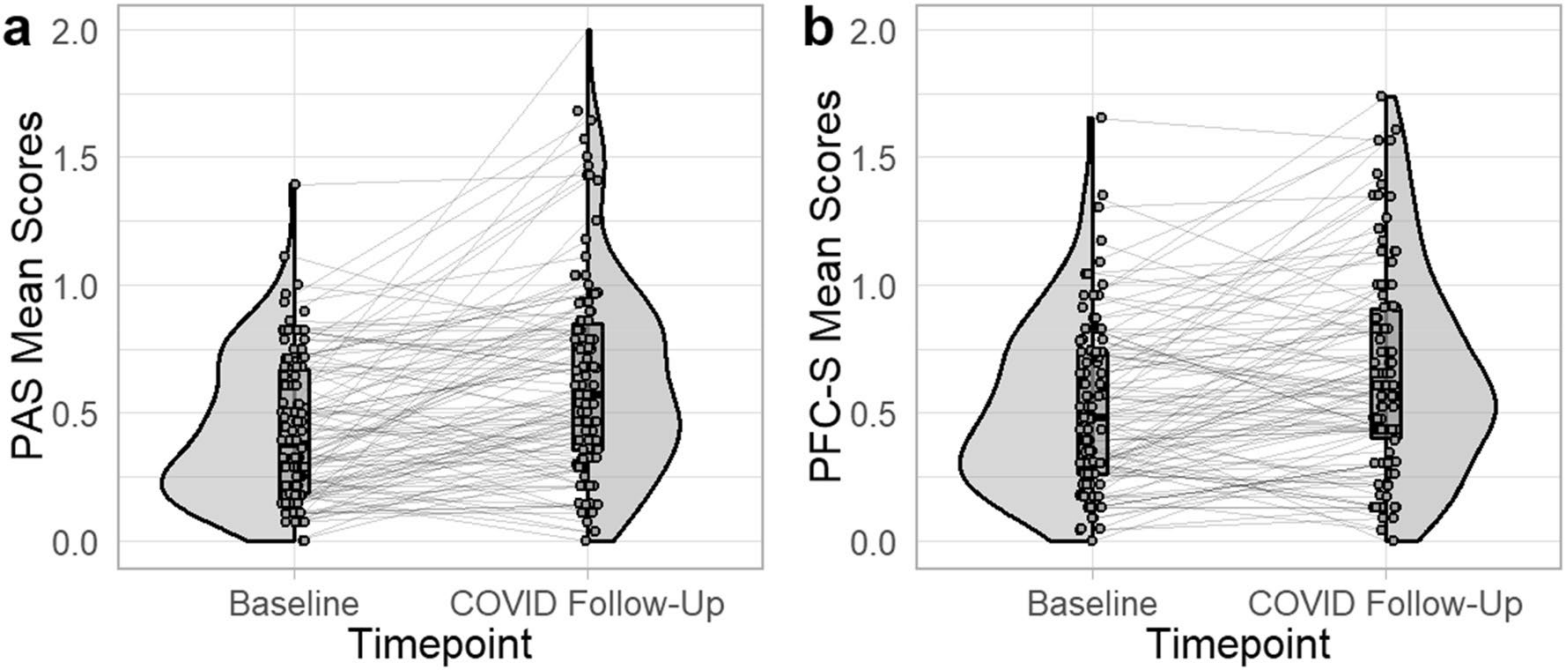
Distributions of mean anxiety and depression symptom severity across timepoints. *N* = 106. The distributions of (**a**) anxiety and (**b**) depression symptom severity before the pandemic and at the COVID follow-up timepoints are depicted here. Participants are represented by dots connected by lines across timepoints. Box plots show the mean and limits of the first and third quartiles. Half violin plots show the distributions of the data based on percentage represented at each point, across the full sample. PAS = Preschool Anxiety Scale, PFC-S = Preschool Feelings Checklist – Scale.

Higher levels of both child anxiety, *t*(105) = 6.11, *p* < 0.001, Cohen’s *d* = 0.59, and depression, *t*(105) = 4.62, *p* < 0.001, Cohen’s *d* = 0.45, severities were reported at the COVID follow-up when compared to their baseline visit. There was wide variability in symptom severity change (Fig. 1).

### Multivariate outliers

For each of the main models of interest, six outliers were identified and removed from the sample (*n* = 100). Four of these six participants were considered outliers in both models.

### Anxiety symptom severity during COVID-19

**Figure 2:**
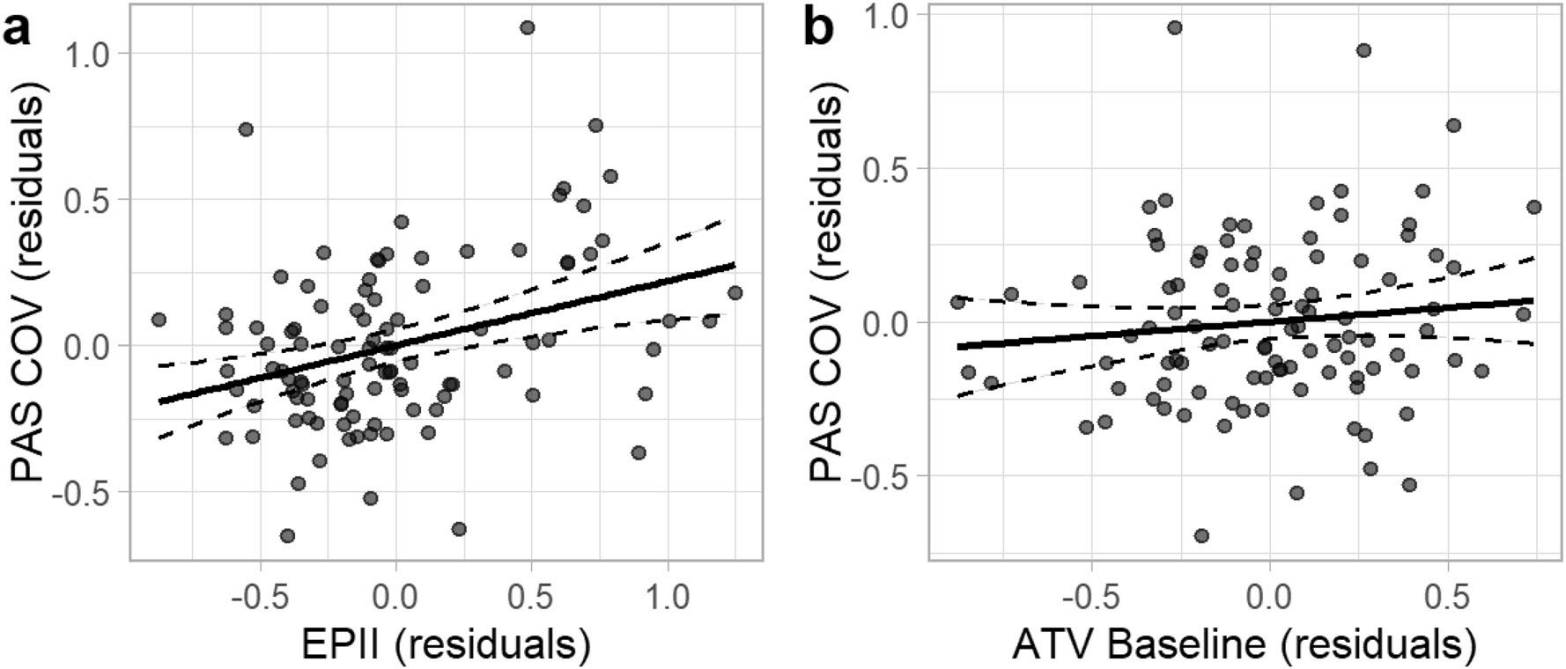
Partial regression plots of specific associations between anxiety symptom severity during the first two months of the pandemic and COVID-19 family stressors and pre-pandemic authoritative parenting style. *Note*. *N* = 100. The specific associations between anxiety symptom severity during the pandemic and (**a**) COVID-19 family stressors and (**b**) pre-pandemic authoritative parenting style are depicted here. Each dot represents an individual participant. Residual scores were estimated for each measure after controlling for relevant covariates. Dashed lines represent 95% confidence intervals. COV = COVID Follow-Up, PAS = Preschool Anxiety Scale, EPII = Epidemic-Pandemic Impacts Inventory, ATV = Parenting Practices Questionnaire, Authoritative Style.

Results are presented in Table 3. After controlling for pre-pandemic child anxiety symptom severity, parent NA, and concurrent depression symptom severity, *R*^2^ = 0.39, Adj. *R*^2^ = 0.37, *F*(3, 96) = 20.24, *p* < 0.001, the addition of the COVID-19 family stressors variable led to a statistically significant increase in the proportion of variation explained in child anxiety symptom severity during the pandemic, Δ*R*^2^ = 0.06, *F*(1, 95) = 10.40, *p* = 0.002. In step three, including pre-pandemic authoritative style scores did not lead to a statistically significant increase in the proportion of variation explained, Δ*R*^2^ = 0.01, *F*(1, 94) = 1.41, *p* = 0.238. The final model containing all five predictors explained a significant proportion of the variation in child anxiety symptom severity during the early stages of the pandemic, *R*^2^ = 0.45, Adj. *R*^2^ = 0.43, *F*(5, 94) = 15.76, *p* < 0.001. Results suggest that anxiety symptom severity during the pandemic is positively associated with COVID-19 stressor impact, *b* = 0.20, b* = 0.27, *t* = 3.13, *p* = 0.002, but not significantly associated with pre-pandemic endorsement of an authoritative style of parenting, *b* = 0.10, b* = 0.10, *t* = 1.19, *p* = 0.238 (Fig. 2). Results were robust to all sensitivity analyses (see Supplementary Materials).

**Table 3:** Hierarchical regression results, anxiety severity during the COVID pandemic.

Predictor	*b*	b*	SE	rSE	Fit	Difference
Step 1						
Intercept	0.13	0.00	0.07	0.07		
PAS T1	0.36	0.25**	0.13	0.12		
PFC-S T2	0.37	0.40***	0.08	0.09		
P-PANAS NA T1	0.13	0.18*	0.06	0.06		
					*R*^2^ = .39** Adj. *R*^2^ = .37	*F*(3, 96) = 20.24***
Step 2						
Intercept	−0.04	0.00	0.09	0.09		
PAS T1	0.41	0.29**	0.12	0.12		
PFC-S T2	0.26	0.28**	0.09	0.09		
P-PANAS NA T1	0.09	0.12	0.06	0.07		
EPII T2	0.21	0.28**	0.06	0.07		
					*R*^2^ = .45** Adj. *R*^2^ = .42	*F*(1, 95) = 10.40, Δ *R*^2^ = .06**
Step 3						
Intercept	−0.36	0.00	0.29	0.29		
PAS T1	0.40	0.28**	0.12	0.12		
PFC-S T2	0.30	0.32**	0.09	0.10		
P-PANAS NA T1	0.10	0.13	0.06	0.07		
EPII T2	0.20	0.27**	0.06	0.07		
PPQ—ATV T1	0.10	0.10	0.08	0.09		
					*R*^2^ = .46** Adj. *R*^2^ = .43	*F*(1, 94) = 1.41, Δ *R*^2^ = .01

*N* = 100. *b* = unstandardized coefficient, b* = standardized coefficient, SE = standard error, rSE = cluster-robus standard error, T1 = baseline, T2 = COVID Follow-Up. EPII = Epidemic-Pandemic Impacts Inventory, PAS = Preschool Anxiety Scale, PFC-S = Preschool Feelings Checklist-Scale, ATV = Parenting Practices Questionnaire, Authoritative Style. P—PANAS NA = Positive and Negative Affect Schedule, Negative Affect (Parent).

### Depression symptom severity during COVID-19

**Figure 3:**
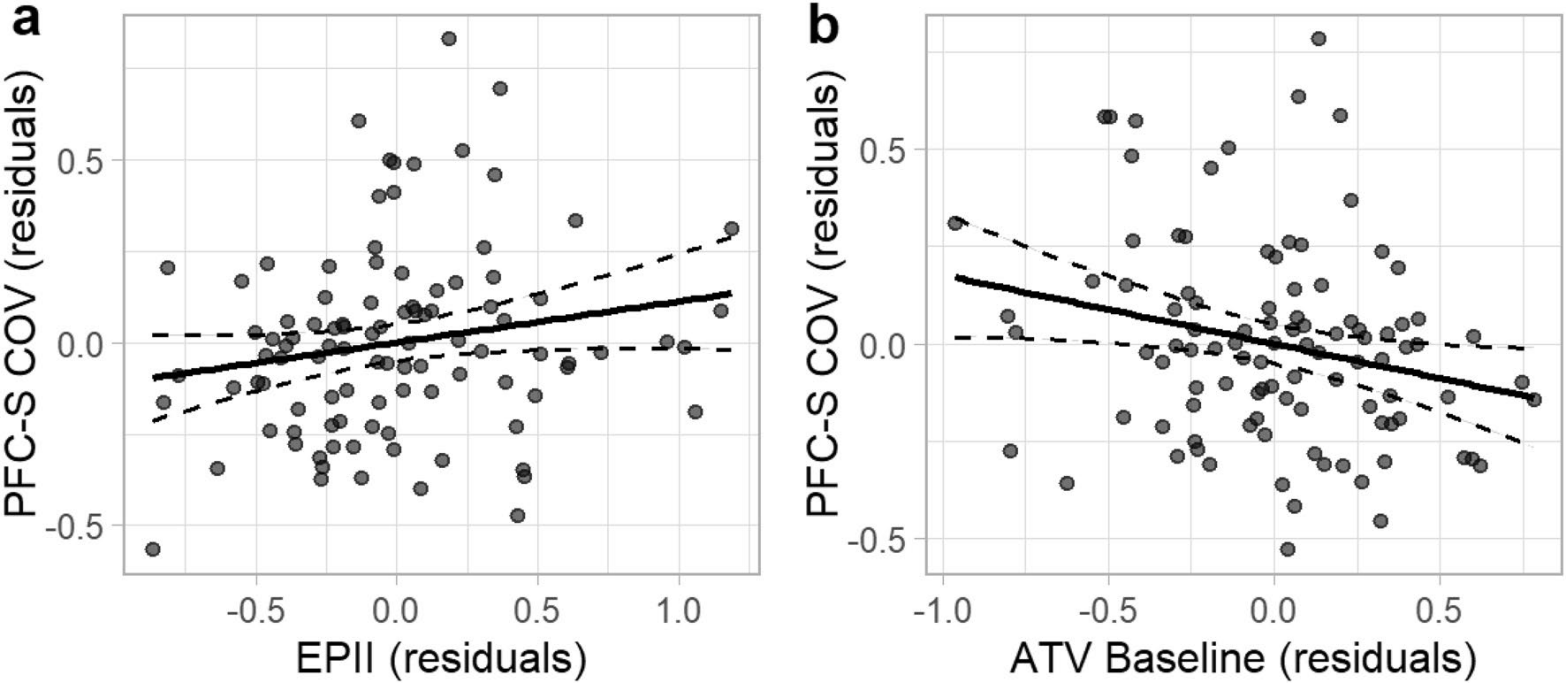
Partial regression plots of specific associations between depression symptom severity during the first two months of the pandemic and COVID-19 family stressors and pre-pandemic authoritative parenting style. *Note*. *N* = 100. The specific associations between depression symptom severity during the pandemic and (**a**) COVID-19 family stressors and (**b**) pre-pandemic authoritative parenting style are depicted here. Each dot represents an individual participant. Residual scores were estimated for each measure after controlling for relevant covariates. Dashed lines represent 95% confidence intervals. COV = COVID Follow-Up, PFC-S = Preschool Feelings Checklist-Scale, EPII = Epidemic-Pandemic Impacts Inventory, ATV = Parenting Practices Questionnaire, Authoritative Style.

Results are presented in Table 4. After controlling for pre-pandemic child depression symptom severity, parent NA, and concurrent anxiety symptom severity, *R*^2^ = 0.52, Adj. *R*^2^ = 0.51, *F*(3, 96) = 35.36, *p* < 0.001, the addition of the COVID-19 family stressors variable did not lead to a statistically significant increase in the proportion of variation explained in child depression symptom severity during the pandemic, Δ*R*^2^ = 0.02, *F*(1, 95) = 2.90, *p* = 0.092. This suggests that COVID-19 family stressors did not explain unique variation in depression symptom severity during the early months of the pandemic. In step three, the addition of the endorsement of a pre-pandemic authoritative style of parenting led to a statistically significant increase in the proportion of variation explained, Δ*R*^2^ = 0.02, *F*(1, 94) = 5.46, *p* = 0.022. The final model containing all five predictors explained a significant proportion of the observed variation in child depression symptom severity during the early stages of the pandemic, *R*^2^ = 0.56, Adj. *R*^2^ = 0.54, *F*(5, 94) = 24.35, *p* < 0.001. Results suggest that depression symptom severity during pandemic onset is negatively associated with the endorsement of an authoritative parenting style, *b* = −0.18, b* = −0.16, *t* = -2.34, *p* = 0.022, but not significantly associated with the impact of COVID-19 family stressors, *b* = 0.11, b* = 0.14, *t* = 1.86, *p* = 0.065 (Fig. 3). All except one of these results were robust to sensitivity analyses (see Supplementary Materials). When statistical outliers were included, the impact of COVID-19 family stressors was independently associated with and explained a unique proportion of the variation in children’s depression symptom severity during the pandemic.

**Table 4:** Hierarchical regression results, depression severity during the COVID pandemic.

Predictor	*b*	b*	SE	rSE	Fit	Difference
Step 1						
Intercept	0.08	0.00	0.07	0.06		
PFC-S T1	0.64	0.51***	0.09	0.08		
PAS T2	0.41	0.40***	0.08	0.09		
P-PANAS NA T1	−0.04	−0.05	0.06	0.06		
					*R*^2^ = .52** Adj. *R*^2^ = .51	*F*(3, 96) = 35.36***
Step 2						
Intercept	−0.01	0.00	0.08	0.07		
PFC-S T1	0.63	0.50***	0.09	0.08		
PAS T2	0.36	0.35***	0.08	0.09		
P-PANAS NA T1	−0.04	−0.06	0.06	0.06		
EPII T2	0.10	0.13	0.06	0.05		
					*R*^2^ = .54** Adj. *R*^2^ = .52	*F*(1, 95) = 2.90, Δ *R*^2^ = .02
Step 3						
Intercept	0.55	0.00*	0.29	0.23		
PFC-S T1	0.59	0.47***	0.12	0.12		
PAS T2	0.34	0.33***	0.09	0.10		
P-PANAS NA T1	−0.04	−0.05	0.06	0.07		
EPII T2	0.11	0.14	0.06	0.07		
PPQ – ATV T1	−0.18	−0.16*	0.08	0.09		
					*R*^2^ = .56** Adj. *R*^2^ = .54	*F*(1, 94) = 5.46, Δ*R*^2^ = .02*

*N* = 100. *b* = unstandardized coefficient, b* = standardized coefficient, SE = standard error, rSE = cluster-robust standard error, T1 = baseline, T2 = COVID Follow-Up. EPII = Epidemic-Pandemic Impacts Inventory, PAS = Preschool Anxiety Scale, PFC-S = Preschool Feelings Checklist-Scale, ATV = Parenting Practices Questionnaire, Authoritative Style. P—PANAS NA = Positive and Negative Affect Schedule, Negative Affect (Parent).

## Discussion

This study investigated whether anxiety and depression symptoms in young children were altered after the onset of COVID-19 pandemic-related changes (e.g., changes in daily life routines and community access). It also tested whether changes in child depression and anxiety symptom severity within the first two months of COVID-19 school closures and other related changes were independently associated with COVID-19 family stressors and with the enduring effects of authoritative parenting style prior to the pandemic. Results suggest that young children exhibited elevated depression and anxiety symptom severity following the onset of COVID-19 restrictions. They also suggest that more COVID-19 family-related stress at this time was associated with increases in child anxiety. Conversely, COVID-19 family-related stress was not associated with increases in depression symptom severity. This result was sensitive to statistical outliers (see Supplementary Materials). Finally, higher authoritative parenting style scores prior to the pandemic were found to be associated with lower depression, but not anxiety, symptom severity in children following the onset of the pandemic. Importantly, the current results remained stable when controlling for income-to-needs, pre-existing negative life stressors, and length of time between data collection timepoints in separate sensitivity analyses.

As predicted, the current study found that children’s anxiety symptom severity increased in the early stage of the pandemic. This finding reflects previous literature showing the prevalence of, and increases in, youth anxiety at the beginning of COVID-19^6,8,9,37^. Furthermore, we found that increased impact of COVID-19 family stressors heightened young children’s anxiety symptom severity at the onset of the pandemic, extending previous research in older children by showing an association between COVID-19 family stressors and anxiety symptoms during early-middle childhood^34^. One mechanism that may be underlying these findings is that exposure to COVID-19-related stressors could have increased children’s intolerance of uncertainty (IU). IU can manifest as negative beliefs and emotional reactivity about the future^62^. IU has been linked to child, youth, and adult anxiety^63–65^ and children’s distressing COVID-19 thoughts and behaviors during the first 18 months of the pandemic^66^, emphasizing a potential relationship between IU at the onset of COVID-19 and anxiety in children. It is possible that early pandemic-related stressors increased our sample’s intolerance of uncertainty, which heightened the severity of their anxiety symptoms. The current study is unable to inform this question due to the lack of research on this age group. Thus, future work should investigate the relationships between young children’s pandemic-related IU, family stressors, and anxiety symptoms to understand the mechanisms that led to increases in young children’s anxiety symptoms.

We found that children’s depression symptom severity increased at the beginning of the pandemic, but this change was not related to the early, acute impact of COVID-19 family stressors. This finding differs from previous research showing significant associations between youth’s depression symptoms and COVID-19 stressors^8,34–38^. It is important to note, however, that a significant association was found between child depression symptom severity and acute COVID-19 family impacts in a sensitivity analysis in which statistical outliers were retained (see Supplementary Materials). Nonetheless, it is possible that early on-going pandemic-related family stressors as measured in this study (e.g., economic burden) did not exacerbate children’s depression symptom severity beyond the impact of other factors that increased children’s depression symptoms at the onset of COVID-19. For example, recent literature has shown associations between children’s depression symptoms and the initial impact of diminished social interaction and increased loneliness instilled by school closures and stay-at-home orders^1,7,33,43,67^. Although the current study cannot inform the sample’s experience of loneliness at the onset of COVID-19, future work should address how child-specific experiences of constructs like loneliness and COVID-19 stressors may differentially account for any changes in their depression symptoms during COVID-19.

We considered pre-pandemic authoritative parenting, known to mitigate long-term effects of family adversity on child adjustment and have longitudinal benefits on children’s mental health^11,26,28,29,68,69^, a proxy for the support children received at the onset of COVID-19. Therefore, we expected pre-pandemic authoritative parenting to have a lasting effect on children's internalizing symptoms through the onset of the pandemic. We found that greater pre-pandemic authoritative parenting style scores was associated with diminished depression symptom severity early in the pandemic, which is in line with previous research^24,25,70–72^. This finding extends work showing concurrent negative associations between child internalizing symptoms and supportive parenting during COVID-19^30,34,37,73^ by (a) demonstrating the enduring influence of pre-pandemic authoritative parenting and (b) using a sample of younger children. Studies showing inverse associations between specific dimensions of authoritative parenting (e.g., parental warmth, low psychological control) and depression symptoms (e.g., loneliness) may inform the mechanisms underlying these results^74–76^. As pandemic-related stressors emerged, authoritative parenting approaches may have buffered the exacerbation of socially-relevant symptoms of depression^1,43,67^. Future research can continue to probe these relationships and elucidate influences of other parenting styles.

Conversely, we found that pre-pandemic authoritative parenting style did not significantly predict children’s anxiety symptom change in the early stages of the pandemic. This is in contrast to literature showing relationships between authoritative parenting and lower anxiety symptoms for youth in general^24,25^ and during COVID-19’s lockdown^30,31^. Measuring parenting style prior to the pandemic rather than concurrently with children’s symptomatology during the pandemic may help explain this discrepancy. Additionally, different studies may be measuring distinct parenting dimensions. Situational parenting practices, like emotion-coaching and parent–child discussions, have previously been associated with decreases in children’s anxiety symptoms during COVID-19^34–37^. The broader emotional climate instilled by parenting styles may be a different construct and have distinct impacts on children compared to situational parenting practices^21,77^. It is possible that rather than relying on a previously established authoritative parenting style and its supportive climate to decrease children’s anxiety, as supposed in our study, children benefited more from situational parenting practices, as measured in previous work, that provided coping strategies in the face of stressors^78^. Future research should investigate the differential effects and interactions of authoritative parenting style with advantageous parenting practices during COVID-19 to support the development of healthy parenting in preparation for future collective traumas and/or large-scale societal stressors.

Findings from the current study suggest that the increases in depression symptoms during acute and unexpected significant stressors like COVID-19 are attenuated for children with parents who endorse using an authoritative parenting style prior to the event. As a result, findings begin to fill the gap in existing literature on parenting style and child mental health in the context of large-scale stressors and suggest that an authoritative parenting style established prior to the onset of the collective trauma potentially protects children’s socioemotional functioning during this context. Additionally, and importantly, the positive effects of authoritative parenting on child depressive symptom levels following COVID-19 in the current study remained significant after controlling for income-to-needs and prior negative life events in sensitivity analyses (see Supplementary Tables S3-S6). And, as such, the current study also provides a novel extension of existing parenting research by suggesting that the positive child mental health effects of an authoritative parenting style prior to significant global disruptions of family and community life are unlikely to vary based on family resources and/or prior levels of stress. While future research will be necessary to replicate these findings and extend them into more representative samples before any suggestions on best familial practices are given to the public, the current study nevertheless provides an important step forward in our understanding of how an authoritative parenting style can have positive effects on child mental health during future significant and acute global stressors such as COVID-19.

There are several limitations to consider when interpreting these results. First, the data on anxiety and depression symptoms in children were collected through parent reports rather than direct observation. However, the PFC-S demonstrates strong concurrent and predictive validity^56^, and the PAS shows reasonable construct validity^53^. Second, parents’ PPQ responses may have been influenced by social desirability, which could have artificially skewed their endorsements of authoritative parenting^79^. Third, we were unable to control for parenting style during the pandemic because these data were only collected at baseline. While research shows that estimates of sensitive parenting behaviors remain relatively stable during early childhood^69^, we do not know for certain how styles were affected by pandemic-related stressors. Fourth, we did not control for parents' negative affect during the pandemic. Parent mental health and stress has impacted children during COVID-19^80^, so future work should test whether our results hold when controlling for parental negative affect during the pandemic. One final limitation to the study is its lack of generalizability. Our sample does not match national racial and ethnic demographics and is not reflective of the full range financial stress resulting from COVID-19^81^. Future research should use a similar modeling approach to uncover the relationship between pre-pandemic authoritative parenting style and children’s mental health during the pandemic in populations disproportionately impacted by COVID-19.

## Conclusion

The current study informs the acute effects of the COVID-19 pandemic on young children’s mental health. It suggests that idiosyncratic family stressors negatively impacted the experience and expression of anxiety symptoms by young children during the early stages of COVID-19 restrictions on in-person schooling and community access. It also indicates that an authoritative parenting style may have acted to buffer a child's vulnerability to experiencing increased depression symptoms following the onset of this collective trauma. Although additional public health research is needed prior to providing information to the public on protective factors and best familial practices for future pandemics and/or collective traumas, this study can help inform researchers on the importance of considering the interplay between individual-level family stressors and parent–child relationships when assessing the impact of COVID-19 and other collective traumas on young children’s mental health.

### Supplementary Information

Supplementary Information.

## Data Availability

The datasets generated during and analyzed during the current study are available in the Github repository, https://github.com/nicocamacho94/covid_parentingStyle_dep_anx.
