# Supplementary material for: Associations between pre-pandemic authoritative parenting, pandemic stressors, and children’s depression and anxiety at the initial stage of the COVID-19 pandemic
Source: Sci Rep. 2023 Sep 20;13:15592. doi: 10.1038/s41598-023-42268-x (PMC10511718; doi:10.1038/s41598-023-42268-x)
Supplement: Supplementary file 1 — Supplementary Information. [file 41598_2023_42268_MOESM1_ESM.docx]

Supplementary Materials for

**Associations Between Pre-Pandemic Authoritative Parenting, Pandemic Stressors, and Children’s Depression and Anxiety at the Initial Stage of the COVID-19 Pandemic**

Karina G. Heaton, Nicolas L. Camacho, and Michael S. Gaffrey

Corresponding author:

Karina G. Heaton

Rutgers University, Graduate School of Applied and Professional Psychology

152 Frelinghuysen Rd, Piscataway, NJ 08854

Email: [kh831@gsapp.rutgers.edu](mailto:kh831@gsapp.rutgers.edu)

**Table of Contents**

1. Supplementary Figure 1: Plot of Ages of Child Participants at Both Study Timepoints
2. Supplementary Figure 2: Plot of Dates of Participation at Both Study Timepoints
3. Additional Measures Specifications
4. Details on R packages Used in Analyses
5. Testing Assumptions of Homoscedasticity and Residual Normality in Regression Models
6. Specifications for Sensitivity Analyses
7. Supplementary Table 1: Hierarchical regression results, anxiety severity during the COVID pandemic, outliers included
8. Supplementary Table 2: Hierarchical regression results, depression severity during the COVID pandemic, outliers included
9. Supplementary Table 3: Hierarchical regression results, anxiety severity during the COVID pandemic, ItN as a covariate
10. Supplementary Table 4: Hierarchical regression results, depression severity during the COVID pandemic, ItN as a covariate
11. Supplementary Table 5: Hierarchical regression results, anxiety severity during the COVID pandemic, LEC as a covariate
12. Supplementary Table 6: Hierarchical regression results, depression severity during the COVID pandemic, LEC as a covariate
13. Supplementary Table 7: Hierarchical regression results, anxiety severity during the COVID pandemic, time between baseline and follow-up timepoints as a covariate
14. Supplementary Table 8: Hierarchical regression results, depression severity during the COVID pandemic, time between baseline and follow-up timepoints as a covariate

**Supplementary Figure 1: Plot of Ages of Child Participants at Both Study Timepoints**

**
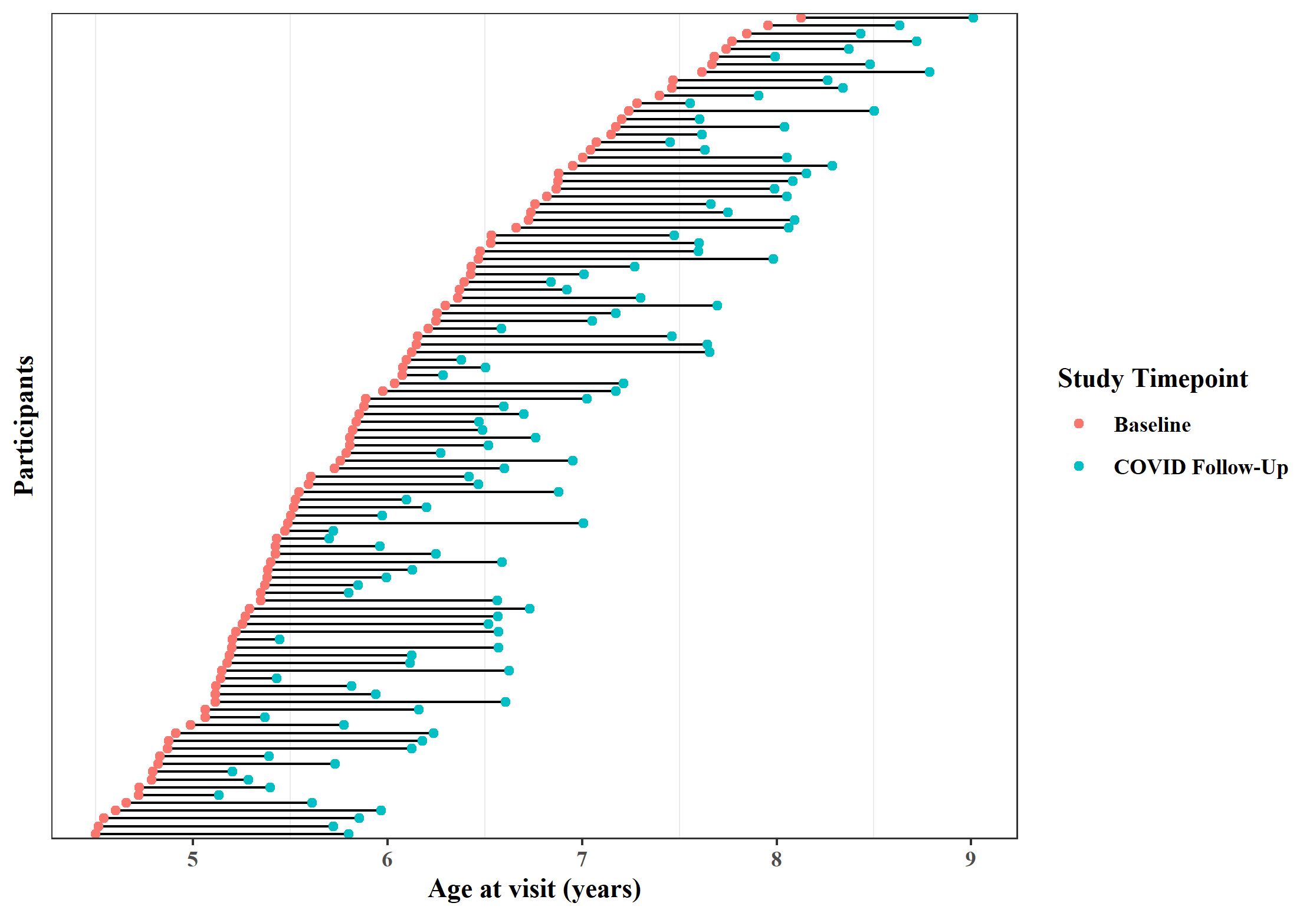
**

**Supplementary Figure 2: Plot of Dates of Participation at Both Study Timepoints**


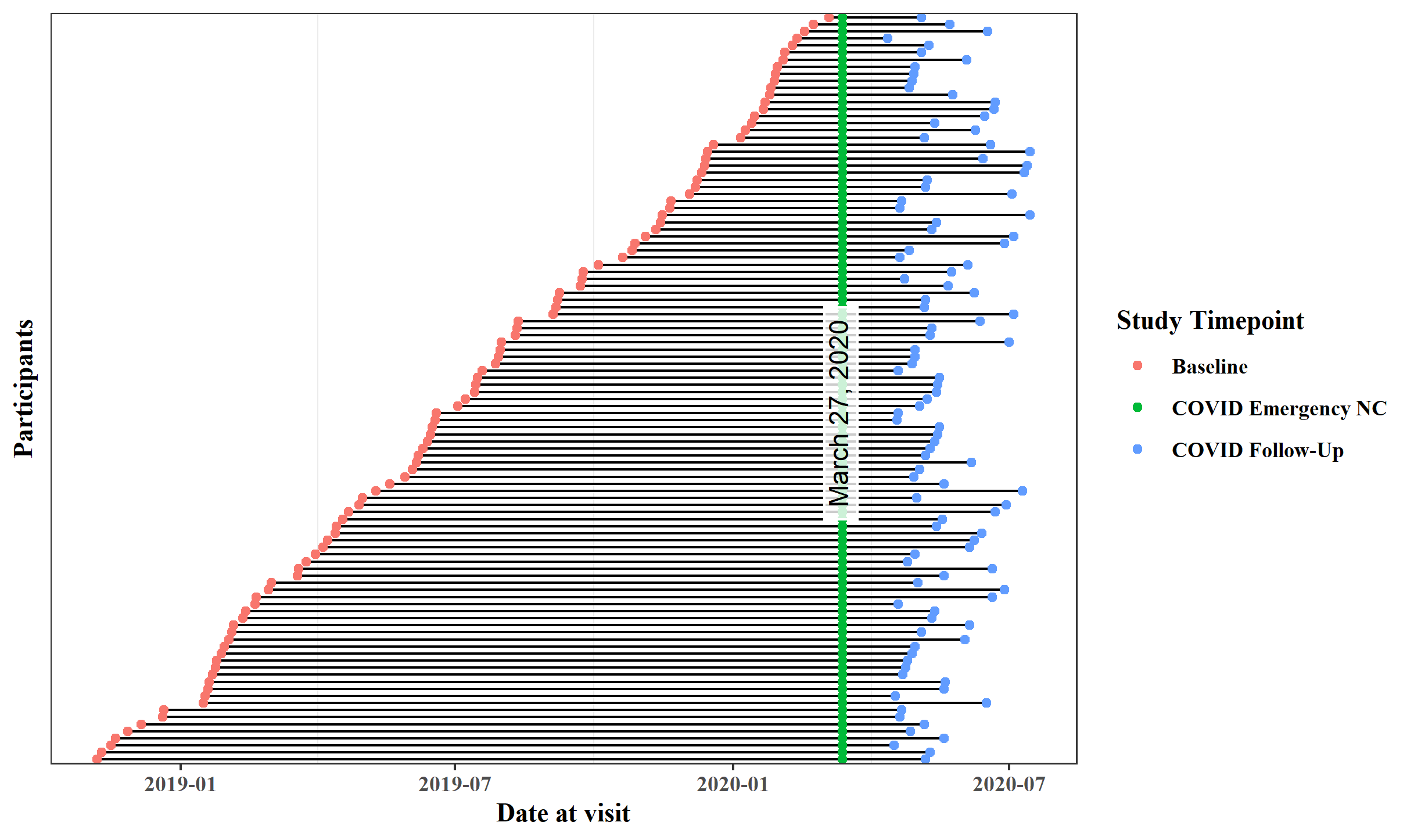


**References**

The R code used to create Supplementary Figures 1 and 2 was written by Paul A. Bloom, PhD, and can be found here: https://github.com/pab2163/amygdala_mpfc_multiverse. The code was edited by author N.L.C. to fit the data in this study.

**Additional Measures Specifications**

**Life Events Checklist** (LEC; [Brand & Johnson, 1982](https://journals.sagepub.com/doi/pdf/10.2466/pr0.1982.50.3c.1274)): During the baseline visit, parents were asked to fill out a checklist that probed whether 39 specific events had never happened to the child, happened in the past 6 months, or happened more than 6 months ago. They were asked to state if they believed the experience of each event was perceived as good or bad by the child. Then, they rated on a Likert scale how much of an effect each experienced event had on their child’s life. The LEC’s effects scale includes options for 0 – No effect, 1 – Some effect, 3 – Moderate effect, and 4 – Great effect.

**Epidemic Pandemic Impact Inventory** (EPII; Grasso et al., 2020): The sections in the EPII measure include Work Employment, Education and Training, Home Life, Economic, Social Activities, Emotional Health and Wellbeing, Quarantine, Infection, Physical Health, and Positive Changes. The inventory contains 92 items. Each question is answered using a Likert scale, including responses for 1 – No negative effect, 2 – Some negative effect, 3 – Moderate negative effect and 4 – Great negative effect. The scale was changed to 0 (No negative effect) – 3 (Great negative effect) to allow for proper data analysis.

**Preschool Anxiety Scale** (PAS; Spence et al., 2001): There are 34 questions in this parent-report scale, measuring children’s symptoms related to generalized anxiety, social anxiety, obsessive compulsive disorder, physical injury, and separation anxiety. For example, some of the questions asked are, “Is afraid of crowded or closed-in places” and “Asks for reassurance when it doesn’t seem necessary.” Each question is answered using a Likert scale, including responses for 0 – Not true at all, 1 – Seldom true, 2 – Sometimes true, 3 – Quite often true, and 4 – Very often true.

**Preschool Feelings Checklist Scale** (PFC-S; Luby et al., 2004): There are 23 questions in this scale asking parents to best describe their child’s behaviors and feelings over the past week. For example, some questions asked are, “Appears sad or says s/he feels sad” and “Seems to feel overly guilty.” Each question is answered using a Likert scale, including responses for 0 – Never, 1 – Seldom true, 2 – Rarely, 3 – Often, and 4 – Most of the time.

**Parenting Practices Questionnaire** (PPQ; Robinson et al., 1995): This measure was given to caregivers only during the baseline visit. There are 62 questions on this scale asking parents to best describe their recruitment of behaviors indicative of three different parenting styles: authoritarian, authoritative, and permissive. This scale consists of items that describe practices that indicate a warm, supportive, and autonomy-granting approach to parenting. Questions asked included “Encourages our child to talk about the child's troubles.” and “Shows sympathy when our child is frustrated or hurt.” Each question is answered using a Likert scale, including responses for 1 – Never, 2 – Once in a while, 3 – About half the time, 4 – Very often, and 5 – Always. The score for each parent’s authoritative parenting style was calculated based on the items that corresponded to this parenting approach.

**Positive and Negative Affect Schedule – Parent** (P-PANAS; [Watson, Clark, and Tellegen, 1988](https://psycnet.apa.org/buy/1988-31508-001)): The negative affect (NA) scale of the PANAS is composed of 10 items that contain one-word adjectives of feelings and emotions associated with negative affect. Parents were asked to rate the extent to which they relate to feeling each item on average. Items included words such as “Guilty”, “Scared”, “Hostile”, “Irritable”, and “Ashamed.” Each item is answered using a Likert scale, including responses for 1 – Very Slightly or Not at all, 2 – A little, 3 – Moderately, 4 – Quite a bit, and 5 – Extremely.

**References**

Brand, A. H., & Johnson, J. H. (1982). Note on reliability of the life events checklist.

*Psychological Reports*, *50* (3, Pt 2), Article 1274. https://doi.org/10.2466/pr0.1982.50.3c.1274

Grasso, D.J., Briggs-Gowan, M.J., Ford, J.D., & Carter, A.S. (2020). The Epidemic – Pandemic

Impacts Inventory (EPII). University of Connecticut School of Medicine.

Luby, J. L., Heffelfinger, A., Koenig-McNaught, A. L., Brown, K., & Spitznagel, E. (2004). The

preschool feelings checklist: A brief and sensitive screening measure for depression in young children. *Journal of the American Academy of Child & Adolescent Psychiatry*, *43*(6), 708–717. https://doi.org/10.1097/01.chi.0000121066.29744.08

Robinson, C. C., Mandleco, B., Olsen, S. F., & Hart, C. H. (1995). Authoritative, authoritarian,

and permissive parenting practices: Development of a new measure. *Psychological Reports*, *77*(3), 819–830. https://doi.org/10.2466/pr0.1995.77.3.819

Spence, S. H., Rapee, R., McDonald, C., & Ingram, M. (2001). The structure of anxiety

symptoms among preschoolers. *Behaviour Research and Therapy*, *39*(11), 1293–1316. https://doi.org/10.1016/s0005-7967(00)00098-x

Watson, D., Clark, L. A., & Tellegen, A. (1988). Development and validation of brief measures

of positive and negative affect: The panas scales. *Journal of Personality and Social Psychology*, *54*(6), 1063–1070. https://doi.org/10.1037/0022-3514.54.6.1063

**R Packages used for Analyses**

The *Hmisc* package ([Harrell & DuPont, 2021](https://cran.r-project.org/web/packages/Hmisc/Hmisc.pdf)) was used to conduct Pearson’s *r* correlations for all variables of main interest to the study. Paired-sample t-tests were conducted using the *rstatix* package (Kassambara, 2021) to test where significant changes were evident in children’s depression and anxiety symptom severity between the baseline and COVID-19 follow-up assessments. The *MASS* package (Ripley et al., 2021) was used to identify model-specific multivariate outliers for the analyses of this study. The Minimum Covariance Determinant estimator was used to identify outliers. The primary analyses of this study, hierarchical linear regressions, were conducted using the *stats* package in R (R Core Team, 2021). To estimate cluster robust standard errors of the regression analyses conducted, we used the *miceadds* package ([Robitzsch, Grund, and Henke, 2021](https://cran.r-project.org/web/packages/miceadds/miceadds.pdf)), which calls on the *sandwich* package to employ Eicker-Huber-White sandwich covariance estimation (Zeleis, 2004; [Zeileis et al., 202](https://cran.r-project.org/web/packages/sandwich/sandwich.pdf)1). All visualizations were created using various functions in the *tidyverse* package (Wickham et al., 2019).

**References**

Harrell, F.E. & DuPont, C. (2021). Hmisc. R package version 4.7-0.

https://CRAN.R-project.org/package=Hmisc

[Kassambara, A. (2021](https://cran.r-project.org/web/packages/rstatix/rstatix.pdf)). Rstatix. R package version 0.7.0.

https://CRAN.R-project.org/package=rstatix

[Ripley, B., Venables, B., Bates, D.M., Hornik, K., Gebhardt, A., & Firth, D. (2021](https://cran.r-project.org/web/packages/MASS/MASS.pdf)). MASS. R

package version 7.3-57. https://CRAN.R-project.org/package=MASS

[R Core Team. (](https://stat.ethz.ch/R-manual/R-devel/library/stats/html/00Index.html)2021). Stats. R package version 4.3.0.

https://stat.ethz.ch/R-manual/R-devel/library/stats/html/00Index.html

[Robitzsch, A., Grund, S., & Henke, T. (2021](https://cran.r-project.org/web/packages/miceadds/miceadds.pdf)). Miceadds. R package version 3.12-26.

https://CRAN.R-project.org/package=miceadds

Wickham H, Averick M, Bryan J, Chang W, McGowan LD, François R, Grolemund G, Hayes

A, Henry L, Hester J, Kuhn M, Pedersen TL, Miller E, Bache SM, Müller K, Ooms J, Robinson D, Seidel DP, Spinu V, Takahashi K, Vaughan D, Wilke C, Woo K, Yutani H (2019). Welcome to the tidyverse. *Journal of Open Source Software*, **4**(43), 1686. doi: 10.21105/joss.01686.

Zeileis, A. (2004). Econometric Computing with HC and HAC Covariance Matrix Estimators.

*Journal of Statistical Software*, 11(10), 1-17. https://doi.org/10.18637/jss.v011.i10.

Zeileis, A., Lumley, T., Graham, N., & Koell, S. (2021). Sandwich. R package version. 3.0-1.

https://sandwich.r-forge.r-project.org/articles/sandwich.html

**Testing Assumptions of Homoscedasticity and Residual Normality in Regression Models**

To ensure that ordinary least squares (OLS) regression could be used to analyze the data in this study, the final models (Step 3) that were included in the main hierarchical regression analyses without outliers were tested for assumptions of residual homoscedasticity and normality. To test for residual homoscedasticity, the Breusch-Pagan (BP) Test was conducted. To test for residual normality, the Kolmogorov-Smirnov (KS) Test was conducted. The final model predicting depression severity during the pandemic met residual homoscedasticity and normality assumptions (BP statistic = 9.06, df = 5, *p* = .11; KS statistic = 0.11, *p* = .21), as did the final model predicting anxiety severity during the pandemic (BP statistic = 10.68, *p* = .06; KS statistic = 0.09, *p* = .43). In each of these tests, p > 0.05 indicates that the null hypotheses cannot be rejected, such that (1) the models are homoscedastic and (2) the residuals are normal, making OLS regression justifiable without further corrective actions.

**Specifications for Sensitivity Analyses**

Various sensitivity analyses were conducted. First, both models were run on the sample of participants that included participants considered multivariate outliers (i.e., MCD method) that were excluded in the main analyses. In separate analyses without outliers, the income-to-needs ratio variable was included as a covariate in step one. Despite this variable having low variability in this sample, this analysis was done due to evidence suggesting that COVID-19 stressors can impact families of different socioeconomic statuses to varying degrees ([Li et al., 2021](https://www.sciencedirect.com/science/article/pii/S0165032721002342?casa_token=49z5bcUF42kAAAAA:hC6gdDbLokLUe9lp0ntv7RoUuC8-ghIyzCpSbjhfHihOeiq_z3nu110ex5m8TJgRF0Y0BsnN#fig0002)). Similarly, in separate analyses without outliers, the negative life stressors variable was included as a covariate in step one to account for the influence of pre-pandemic negative life stressors that may have lasting effects on children’s internalizing symptoms ([Gotlib et al., 2021](https://internal-journal.frontiersin.org/articles/10.3389/fpsyg.2020.603748/full); Foster et al., 2022). The same analysis was conducted with the number of months between study timepoints as a covariate. Outlier detection and removal was also conducted for the sensitivity analyses including ItN (anxiety model, *N* = 96; depression model, *N* = 94), LEC (anxiety model, *N* = 99; depression model, *N* = 99), and months between timepoints (anxiety model, *N* = 104; depression model, *N* = 102) as covariates.

Finally, since some of our participants are siblings, the violation of sample independence due to commonalities in the familial environment was considered ([McNeish, 2014](http://www.glmj.org/archives/articles/McNeish_v40n1.pdf)). Cluster robust standard errors were calculated to account for family membership. While regression coefficient and R^2^ estimates remain unchanged in this sensitivity analysis, the standard errors provided by this robust estimation approach better reflect the variability in the coefficient estimates after accounting for the clustering in the sample ([McNeish, Stapleton, and Silverman, 2016](https://www.researchgate.net/publication/292091130_On_the_Unnecessary_Ubiquity_of_Hierarchical_Linear_Modeling#fullTextFileContent)).

**References**

Foster, J.C., Cohodes, E.M., Brieant, A.E., McCauley, S., Odriozola, P., Zacharek, S.J., Pierre,

J.C., Hodges, H.R., Kribakaran, S., Haberman, J.T., Holt-Gosselin, B., & Gee, D.G. (in press). Associations between early-life stress exposure and internalizing symptomatology during the COVID-19 pandemic: Assessing the role of neurobehavioral mediators. *Biological Psychiatry Global Open Science*. https://doi.org/10.1016/j.bpsgos.2022.07.006

Gotlib, I., Borchers, L., Chahal, R., Gifuni, A., Teresi, G. I., & Ho, T. C. (2020). Early life stress

predicts depressive symptoms in adolescents during the COVID-19 pandemic: The mediating role of Perceived stress. *Frontiers in Psychology*. *11,* Article e603748. https://doi.org/10.31234/osf.io/4dkaf

Li, W., Wang, Z., Wang, G., Ip, P., Sun, X., Jiang, Y., & Jiang, F. (2021). Socioeconomic inequality in child mental health during the COVID-19 pandemic: First evidence from China. *Journal of Affective Disorders*, *287*, 8–14. https://doi.org/10.1016/j.jad.2021.03.009

McNeish, D. M. (2014). Analyzing Clustered Data with OLS Regression: The Effect of a

Hierarchical Data Structure. *Multiple Linear Regression Viewpoints*, *40*(1), 11–16. http://www.glmj.org/archives/articles/McNeish_v40n1.pdf

McNeish, D., Stapleton, L. M., & Silverman, R. D. (2017). On the unnecessary ubiquity of

hierarchical linear modeling. *Psychological Methods*, *22*(1), 114–140. https://doi.org/10.1037/met0000078

**Supplementary Table 1: Hierarchical regression results, anxiety severity during the COVID pandemic, outliers included**

| Predictor | *b* | *b** | SE | rSE | Fit | Difference |
| --- | --- | --- | --- | --- | --- | --- |
| Step 1 |  |  |  |  |  |  |
| Intercept | 0.14 | 0.00* | 0.07 | 0.07 |  |  |
| PAS T1 | 0.46 | 0.32** | 0.12 | 0.12 |  |  |
| PFC-S T2 | 0.44 | 0.45*** | 0.08 | 0.11 |  |  |
| P-NA T1 | 0.01 | 0.12 | 0.06 | 0.06 |  |  |
|  |  |  |  |  | *R*^2^ = .42**  Adj. *R*^2^ = .40 |  |
| Step 2 |  |  |  |  |  |  |
| Intercept | -0.02 | 0.00 | 0.09 | 0.09 |  |  |
| PAS T1 | 0.53 | 0.37** | 0.12 | 0.13 |  |  |
| PFC-S T2 | 0.33 | 0.33** | 0.09 | 0.09 |  |  |
| P-NA T1 | -0.03 | -0.04 | 0.06 | 0.07 |  |  |
| **EPII T2** | **0.20** | **0.25**** | **0.07** | **0.08** |  |  |
|  |  |  |  |  | *R*^2^ = .47**  Adj. *R*^2^ = .44 | *F*(1, 101) = 9.03,  Δ *R*^2^ = .04** |
| Step 3 |  |  |  |  |  |  |
| Intercept | -0.05 | 0.00 | 0.30 | 0.29 |  |  |
| PAS T1 | 0.53 | 0.37** | 0.12 | 0.13 |  |  |
| PFC-S T2 | 0.33 | 0.34** | 0.09 | 0.10 |  |  |
| P-NA T1 | -0.03 | -0.04 | 0.06 | 0.07 |  |  |
| EPII T2 | 0.20 | 0.25** | 0.07 | 0.08 |  |  |
| **ATV T1** | **0.01** | **0.01** | **0.09** | **0.09** |  |  |
|  |  |  |  |  | *R*^2^ = .46**  Adj. *R*^2^ = .43 | *F*(1, 100) = 0.01,  Δ *R*^2^ = .01 |

*Note.* *N* = 106. *b* = unstandardized coefficient, *b** = standardized coefficient, SE = standard error, rSE = cluster-robust standard error.

**Supplementary Table 2: Hierarchical regression results, depression severity during the COVID pandemic, outliers included**

| Predictor | *b* | *b** | SE | rSE | Fit | Difference |
| --- | --- | --- | --- | --- | --- | --- |
| Step 1 |  |  |  |  |  |  |
| Intercept | 0.06 | 0.00 | 0.07 | 0.06 |  |  |
| PFC-S T1 | 0.60 | 0.48*** | 0.09 | 0.08 |  |  |
| PAS T2 | 0.42 | 0.42*** | 0.07 | 0.07 |  |  |
| P-NA T1 | 0.03 | 0.04 | 0.05 | 0.06 |  |  |
|  |  |  |  |  | *R*^2^ = .55***  Adj. *R*^2^ = .53 |  |
| Step 2 |  |  |  |  |  |  |
| Intercept | -0.04 | 0.00 | 0.08 | 0.07 |  |  |
| PFC-S T1 | 0.58 | 0.46*** | 0.09 | 0.08 |  |  |
| PAS T2 | 0.37 | 0.37*** | 0.07 | 0.07 |  |  |
| P-NA T1 | 0.00 | 0.00 | 0.05 | 0.06 |  |  |
| **EPII T2** | **0.13** | **0.16*** | **0.06** | **0.05** |  |  |
|  |  |  |  |  | *R*^2^ = .57***  Adj. *R*^2^ = .55 | *F*(1, 101) = 4.83,  Δ *R*^2^ = .02* |
| Step 3 |  |  |  |  |  |  |
| Intercept | 0.66 | 0.00** | 0.25 | 0.23 |  |  |
| PFC-S T1 | 0.54 | 0.43*** | 0.09 | 0.08 |  |  |
| PAS T2 | 0.34 | 0.34*** | 0.07 | 0.07 |  |  |
| P-NA T1 | 0.01 | 0.01 | 0.05 | 0.05 |  |  |
| EPII T2 | 0.13 | 0.17* | 0.06 | 0.05 |  |  |
| **ATV T1** | **-0.22** | **-0.19*** | **0.07** | **0.07** |  |  |
|  |  |  |  |  | *R*^2^ = .60***  Adj. *R*^2^ = .58 | *F*(1, 100) = 8.91,  Δ *R*^2^ = .03** |

*Note.* *N* = 106. *b* = unstandardized coefficient, *b** = standardized coefficient, SE = standard error, rSE = cluster-robust standard error.

**Supplementary Table 3: Hierarchical regression results, anxiety severity during the COVID pandemic, ItN as a covariate**

| Predictor | *b* | *b** | SE | rSE | Fit | Difference |
| --- | --- | --- | --- | --- | --- | --- |
| Step 1 |  |  |  |  |  |  |
| Intercept | 0.11 | 0.00 | 0.10 | 0.08 |  |  |
| PAS T1 | 0.45 | 0.33*** | 0.12 | 0.11 |  |  |
| PFC-S T2 | 0.35 | 0.39*** | 0.08 | 0.09 |  |  |
| P-NA T1 | 0.14 | 0.20* | 0.06 | 0.06 |  |  |
| ItN T1 | -0.01 | -0.02 | 0.03 | 0.02 |  |  |
|  |  |  |  |  | *R*^2^ = .45***  Adj. *R*^2^ = .43 |  |
| Step 2 |  |  |  |  |  |  |
| Intercept | -0.07 | 0.00 | 0.11 | 0.09 |  |  |
| PAS T1 | 0.49 | 0.36*** | 0.11 | 0.12 |  |  |
| PFC-S T2 | 0.26 | 0.29** | 0.08 | 0.08 |  |  |
| P-NA T1 | 0.11 | 0.16* | 0.05 | 0.07 |  |  |
| ItN T1 | -0.01 | -0.02 | 0.02 | 0.02 |  |  |
| **EPII T2** | **0.20** | **0.26**** | **0.06** | **0.07** |  |  |
|  |  |  |  |  | *R*^2^ = .51***  Adj. *R*^2^ = .48 | *F*(1, 88) = 9.87,  Δ *R*^2^ = .06** |
| Step 3 |  |  |  |  |  |  |
| Intercept | -0.42 | 0.00 | 0.28 | 0.29 |  |  |
| PAS T1 | 0.48 | 0.35*** | 0.11 | 0.12 |  |  |
| PFC-S T2 | 0.30 | 0.33*** | 0.09 | 0.09 |  |  |
| P-NA T1 | 0.12 | 0.17* | 0.05 | 0.06 |  |  |
| ItN T1 | -0.01 | -0.02 | 0.02 | 0.02 |  |  |
| EPII T2 | 0.20 | 0.25** | 0.06 | 0.07 |  |  |
| **ATV T1** | **0.11** | **0.11** | **0.08** | **0.08** |  |  |
|  |  |  |  |  | *R*^2^ = .52***  Adj. *R*^2^ = .48 | *F*(1, 87) = 1.85,  Δ *R*^2^ = .01 |

*Note.* *N* = 98. Two participants were missing ItN. *b* = unstandardized coefficient, *b** = standardized coefficient, SE = standard error, rSE = cluster-robust standard error.

**Supplementary Table 4: Hierarchical regression results, depression severity during the COVID pandemic, ItN as a covariate**

| Predictor | *b* | *b** | SE | rSE | Fit | Difference |
| --- | --- | --- | --- | --- | --- | --- |
| Step 1 |  |  |  |  |  |  |
| Intercept | -0.00 | 0.00 | 0.10 | 0.09 |  |  |
| PFC-S T1 | 0.67 | 0.49*** | 0.11 | 0.10 |  |  |
| PAS T2 | 0.43 | 0.39*** | 0.09 | 0.10 |  |  |
| P-NA T1 | -0.05 | -0.06* | 0.06 | 0.06 |  |  |
| ItN T1 | 0.02 | 0.07 | 0.03 | 0.03 |  |  |
|  |  |  |  |  | *R*^2^ = .53***  Adj. *R*^2^ = .51 |  |
| Step 2 |  |  |  |  |  |  |
| Intercept | -0.09 | 0.00 | 0.11 | 0.11 |  |  |
| PFC-S T1 | 0.67 | 0.50*** | 0.11 | 0.10 |  |  |
| PAS T2 | 0.37 | 0.34*** | 0.10 | 0.10 |  |  |
| P-NA T1 | -0.05 | -0.06 | 0.06 | 0.06 |  |  |
| ItN T1 | 0.02 | 0.06 | 0.03 | 0.03 |  |  |
| **EPII T2** | **0.11** | **0.12** | **0.07** | **0.06** |  |  |
|  |  |  |  |  | *R*^2^ = .54***  Adj. *R*^2^ = .52 | *F*(1, 86) = 2.09,  Δ *R*^2^ = .01 |
| Step 3 |  |  |  |  |  |  |
| Intercept | 0.65 | 0.00* | 0.28 | 0.26 |  |  |
| PFC-S T1 | 0.63 | 0.46*** | 0.11 | 0.10 |  |  |
| PAS T2 | 0.36 | 0.33*** | 0.09 | 0.10 |  |  |
| P-NA T1 | -0.05 | -0.06 | 0.06 | 0.06 |  |  |
| ItN T1 | 0.02 | 0.05 | 0.03 | 0.03 |  |  |
| EPII T2 | 0.10 | 0.11 | 0.07 | 0.06 |  |  |
| **ATV T1** | **-0.22** | **-0.20**** | **0.08** | **0.07** |  |  |
|  |  |  |  |  | *R*^2^ = .58***  Adj. *R*^2^ = .55 | *F*(1, 85) = 8.00,  Δ *R*^2^ = .04** |

*Note.* *N* = 98. Two participants were missing ItN. *b* = unstandardized coefficient, *b** = standardized coefficient, SE = standard error, rSE = cluster-robust standard error.

**Supplementary Table 5: Hierarchical regression results, anxiety severity during the COVID pandemic, LEC as a covariate**

| Predictor | *b* | *b** | SE | rSE | Fit | Difference |
| --- | --- | --- | --- | --- | --- | --- |
| Step 1 |  |  |  |  |  |  |
| Intercept | 0.09 | 0.00 | 0.07 | 0.07 |  |  |
| PAS T1 | 0.48 | 0.34*** | 0.12 | 0.13 |  |  |
| PFC-S T2 | 0.40 | 0.42*** | 0.08 | 0.09 |  |  |
| P-NA T1 | 0.06 | 0.09 | 0.06 | 0.06 |  |  |
| LEC T1 | 0.03 | 0.05 | 0.04 | 0.04 |  |  |
|  |  |  |  |  | *R*^2^ = .47***  Adj. *R*^2^ = .45 |  |
| Step 2 |  |  |  |  |  |  |
| Intercept | -0.09 | 0.00 | 0.08 | 0.08 |  |  |
| PAS T1 | 0.55 | 0.40*** | 0.11 | 0.12 |  |  |
| PFC-S T2 | 0.29 | 0.31*** | 0.08 | 0.08 |  |  |
| P-NA T1 | 0.01 | 0.02 | 0.06 | 0.06 |  |  |
| LEC T1 | 0.01 | 0.01 | 0.04 | 0.04 |  |  |
| **EPII T2** | **0.22** | **0.29***** | **0.06** | **0.06** |  |  |
|  |  |  |  |  | *R*^2^ = .53***  Adj. *R*^2^ = .51 | *F*(1, 93) = 12.91,  Δ *R*^2^ = .06*** |
| Step 3 |  |  |  |  |  |  |
| Intercept | -0.35 | 0.00 | 0.26 | 0.28 |  |  |
| PAS T1 | 0.54 | 0.39*** | 0.11 | 0.12 |  |  |
| PFC-S T2 | 0.32 | 0.34*** | 0.08 | 0.09 |  |  |
| P-NA T1 | 0.02 | 0.02 | 0.06 | 0.06 |  |  |
| LEC T1 | 0.01 | 0.01 | 0.04 | 0.04 |  |  |
| EPII T2 | 0.22 | 0.28*** | 0.06 | 0.06 |  |  |
| **ATV T1** | **0.08** | **0.08** | **0.08** | **0.08** |  |  |
|  |  |  |  |  | *R*^2^ = .54***  Adj. *R*^2^ = .51 | *F*(1, 92) =1.05,  Δ *R*^2^ = .01 |

*Note.* *N* = 100. *b* = unstandardized coefficient, *b** = standardized coefficient, SE = standard error, rSE = cluster-robust standard error.

**Supplementary Table 6: Hierarchical regression results, depression severity during the COVID pandemic, LEC as a covariate**

| Predictor | *b* | *b** | SE | rSE | Fit | Difference |
| --- | --- | --- | --- | --- | --- | --- |
| Step 1 |  |  |  |  |  |  |
| Intercept | 0.09 | 0.00 | 0.07 | 0.06 |  |  |
| PFC-S T1 | 0.65 | 0.53*** | 0.09 | 0.08 |  |  |
| PAS T2 | 0.35 | 0.35*** | 0.08 | 0.08 |  |  |
| P-NA T1 | -0.01 | -0.02 | 0.06 | 0.05 |  |  |
| LEC T1 | -0.01 | -0.01 | 0.04 | 0.05 |  |  |
|  |  |  |  |  | *R*^2^ = .52***  Adj. *R*^2^ = .50 |  |
| Step 2 |  |  |  |  |  |  |
| Intercept | 0.00 | 0.00 | 0.08 | 0.07 |  |  |
| PFC-S T1 | 0.65 | 0.53*** | 0.09 | 0.08 |  |  |
| PAS T2 | 0.30 | 0.30*** | 0.09 | 0.08 |  |  |
| P-NA T1 | -0.02 | -0.03 | 0.06 | 0.05 |  |  |
| LEC T1 | -0.01 | -0.02 | 0.04 | 0.05 |  |  |
| **EPII T2** | **0.11** | **0.14** | **0.06** | **0.05** |  |  |
|  |  |  |  |  | *R*^2^ = .53***  Adj. *R*^2^ = .51 | *F*(1, 93) = 3.05,  Δ *R*^2^ = .01 |
| Step 3 |  |  |  |  |  |  |
| Intercept | 0.49 | 0.00 | 0.25 | 0.23 |  |  |
| PFC-S T1 | 0.61 | 0.50*** | 0.09 | 0.08 |  |  |
| PAS T2 | 0.30 | 0.29*** | 0.09 | 0.09 |  |  |
| P-NA T1 | -0.02 | -0.02 | 0.06 | 0.05 |  |  |
| LEC T1 | -0.01 | -0.02 | 0.04 | 0.05 |  |  |
| EPII T2 | 0.12 | 0.15 | 0.06 | 0.05 |  |  |
| **ATV T1** | **-0.15** | **-0.15*** | **0.08** | **0.07** |  |  |
|  |  |  |  |  | *R*^2^ = .55  Adj. *R*^2^ = .53 | *F*(1, 92) = 4.17,  Δ *R*^2^ = .0.02* |

*Note.* *N* = 100. *b* = unstandardized coefficient, *b** = standardized coefficient, SE = standard error, rSE = cluster-robust standard error.

**Supplementary Table 7: Hierarchical regression results, anxiety severity during the COVID pandemic, time between baseline and follow-up timepoints as a covariate**

| Predictor | *b* | *b** | SE | rSE | Fit | Difference |
| --- | --- | --- | --- | --- | --- | --- |
| Step 1 |  |  |  |  |  |  |
| Intercept | 0.18 | 0.00 | 0.10 | 0.09 |  |  |
| PAS T1 | 0.43 | 0.30*** | 0.12 | 0.13 |  |  |
| PFC-S T2 | 0.38 | 0.40*** | 0.08 | 0.09 |  |  |
| P-NA T1 | 0.05 | 0.07 | 0.06 | 0.06 |  |  |
| Months, T1-T2 | -0.00 | -0.03 | 0.01 | 0.01 |  |  |
|  |  |  |  |  | *R*^2^ = .38***  Adj. *R*^2^ = .36 |  |
| Step 2 |  |  |  |  |  |  |
| Intercept | 0.03 | 0.00 | 0.12 | 0.09 |  |  |
| PAS T1 | 0.47 | 0.34*** | 0.12 | 0.13 |  |  |
| PFC-S T2 | 0.30 | 0.32*** | 0.09 | 0.09 |  |  |
| P-NA T1 | 0.01 | 0.01 | 0.06 | 0.06 |  |  |
| Months, T1-T2 | -0.00 | -0.01 | 0.01 | 0.01 |  |  |
| **EPII T2** | **0.17** | **0.22*** | **0.07** | **0.07** |  |  |
|  |  |  |  |  | *R*^2^ = .42***  Adj. *R*^2^ = .39 | *F*(1, 98) = 6.54,  Δ *R*^2^ = .04* |
| Step 3 |  |  |  |  |  |  |
| Intercept | -0.14 | 0.00 | 0.31 | 0.33 |  |  |
| PAS T1 | 0.47 | 0.33*** | 0.12 | 0.13 |  |  |
| PFC-S T2 | 0.32 | 0.34** | 0.09 | 0.10 |  |  |
| P-NA T1 | 0.01 | 0.01 | 0.06 | 0.06 |  |  |
| Months, T1-T2 | -0.00 | -0.01 | 0.01 | 0.01 |  |  |
| EPII T2 | 0.16 | 0.22* | 0.07 | 0.08 |  |  |
| **ATV T1** | **0.05** | **0.05** | **0.09** | **0.09** |  |  |
|  |  |  |  |  | *R*^2^ = .42***  Adj. *R*^2^ = .39 | *F*(1, 97) =0.34,  Δ *R*^2^ = .00 |

*Note.* *N* = 104. *b* = unstandardized coefficient, *b** = standardized coefficient, SE = standard error, rSE = cluster-robust standard error.

**Supplementary Table 8: Hierarchical regression results, depression severity during the COVID pandemic, time between baseline and follow-up timepoints as a covariate**

| Predictor | *b* | *b** | SE | rSE | Fit | Difference |
| --- | --- | --- | --- | --- | --- | --- |
| Step 1 |  |  |  |  |  |  |
| Intercept | 0.06 | 0.00 | 0.09 | 0.08 |  |  |
| PFC-S T1 | 0.63 | 0.50*** | 0.09 | 0.08 |  |  |
| PAS T2 | 0.42 | 0.43*** | 0.07 | 0.08 |  |  |
| P-NA T1 | -0.04 | -0.05 | 0.06 | 0.05 |  |  |
| Months, T1-T2 | 0.00 | 0.02 | 0.01 | 0.01 |  |  |
|  |  |  |  |  | *R*^2^ = .53***  Adj. *R*^2^ = .52 |  |
| Step 2 |  |  |  |  |  |  |
| Intercept | -0.02 | 0.00 | 0.11 | 0.10 |  |  |
| PFC-S T1 | 0.63 | 0.50*** | 0.09 | 0.08 |  |  |
| PAS T2 | 0.39 | 0.39*** | 0.08 | 0.08 |  |  |
| P-NA T1 | -0.06 | -0.07 | 0.06 | 0.05 |  |  |
| Months, T1-T2 | 0.00 | 0.03 | 0.01 | 0.01 |  |  |
| **EPII T2** | **0.09** | **0.12** | **0.06** | **0.05** |  |  |
|  |  |  |  |  | *R*^2^ = .53***  Adj. *R*^2^ = .51 | *F*(1, 96) = 2.39,  Δ *R*^2^ = .00 |
| Step 3 |  |  |  |  |  |  |
| Intercept | 0.55 | 0.00* | 0.26 | 0.23 |  |  |
| PFC-S T1 | 0.58 | 0.46*** | 0.09 | 0.08 |  |  |
| PAS T2 | 0.36 | 0.37*** | 0.07 | 0.07 |  |  |
| P-NA T1 | -0.05 | -0.06 | 0.06 | 0.05 |  |  |
| Months, T1-T2 | 0.00 | 0.01 | 0.01 | 0.01 |  |  |
| EPII T2 | 0.10 | 0.13 | 0.06 | 0.05 |  |  |
| **ATV T1** | **-0.18** | **-0.17*** | **0.07** | **0.07** |  |  |
|  |  |  |  |  | *R*^2^ = .57  Adj. *R*^2^ = .54 | *F*(1, 95) = 5.61,  Δ *R*^2^ = .0.04* |

*Note.* *N* = 102. *b* = unstandardized coefficient, *b** = standardized coefficient, SE = standard error, rSE = cluster-robust standard error.
